# Effect of a tailor-made hydrotherapy on physical functions in patients after unilateral unicompartmental knee arthroplasty—A feasibility study

**DOI:** 10.1186/s42836-024-00291-x

**Published:** 2025-02-05

**Authors:** Wai-Wang Chau, Mei-Yan Lau, Tsz-Lung Choi, Gloria Yan-Ting Lam, Michael Tim-Yun Ong, Kevin Ki-Wai Ho

**Affiliations:** 1 Department of Orthopaedics and Traumatology, Chinese University of Hong Kong, Hong Kong SAR, China; 2 Department of Orthopaedics and Traumatology, Alice Ho Miu Ling Nethersole Hospital, Hong Kong SAR, China

**Keywords:** Hydrotherapy, Unicompartmental knee arthroplasty, Physical function, Physical therapy, Rehabilitation

## Abstract

**Introduction:**

Unicompartmental knee arthroplasty (UKA) is one of the treatment options for patients whose osteoarthritis involves one out of the 3 compartments. Patients who underwent UKA benefited from shorter hospital stays, better range of motion, and lower risk of postoperative complications compared with patients who underwent total knee arthroplasty (TKA). Hydrotherapy is being introduced complementary to conventional postoperative rehabilitation programs. No report on the use of hydrotherapy evaluating physical functions on patients who underwent UKA leads us to carry out the present study. This is a feasibility study to investigate the effects of hydrotherapy on physical functions in patients after primary unilateral UKA.

**Methods:**

A retrospective cohort study recruited 68 patients who underwent primary unilateral UKA. Nineteen patients were allocated to the hydrotherapy group and 49 patients were in the convention group. Patients in the hydrotherapy group received hydrotherapy and conventional physiotherapy, and the convention group was given conventional physiotherapy only. The primary outcome was Knee Society Function Score (KFS) measured before surgery, six months, and one year after UKA. Self-reported walking tolerance, Timed Up and Go Test (TUGT), and 30-s Chair Stand Test (30CST) were conducted before and after the completion of rehabilitation. Pain and range of motion were also covered.

**Results:**

Hydrotherapy group showed significantly higher KFS at 6 months (*P* = 0.038) and one year (*P* = 0.030) after operation. Range of motion flexion and extension in the hydrotherapy group were significantly improved at postoperative 4 weeks and the last session of rehabilitation. Self-reported walking tolerance in the hydrotherapy group was significantly longer at the last session (*P* = 0.011). No significant difference was found in TUGT, 30CST, and pain between the two groups after rehabilitation. In both groups, all outcomes were significantly better as compared to preoperative findings.

**Conclusion:**

Patients who underwent UKA after hydrotherapy complementary to conventional physiotherapy showed significant improvements in functions, range of motion, and time to tolerating walking before rest. Pain, mobility, balance, leg strength, and endurance were comparable between the two groups. Combination of hydrotherapy with conventional postoperative physiotherapy rehabilitation yielded even better outcomes than conventional physiotherapy alone. Further research with advanced study design, larger sample size and longer follow-up periods for patients who underwent UKA is recommended.

**Trial registration:**

NCT06459960, retrospectively registered on 13.06.2024 (ClinicalTrials.gov).

## Introduction

Osteoarthritis (OA) of the knee is one of the most common chronic degenerative joint diseases, primarily afflicting aging population, limiting joint movement, and causing disability because of pain and stiffness. Prevalence of radiological knee OA increased with age, being 64.1% in those aged 60 and over, and higher in females than in males [1]. Surgery is the subsequent treatment after conservative management fails. Unicompartmental knee arthroplasty (UKA) is one of the treatment options for patients whose osteoarthritis does not involves all compartments [2]. Patients who underwent UKA benefited from shorter hospital stay, better range of motion, and lower risk of postoperative complications, compared with their counterparts who received total knee arthroplasty (TKA) [2]. Our team investigated the correlation between femoral and tibial component axial rotational alignment and functional outcomes of 83 Oxford UKAs received by 67 patients with isolated medial or lateral compartment knee osteoarthritis [3]. We found that femoral component axial rotation between 2° and 6° external rotation, and tibial component axial rotation between 1° and 8° external rotation correlated with significantly better functional scores, with the highest functional scores observed at 3°–4° external rotation for femoral component, and 4°–5° external rotation for tibial component [3].

After knee arthroplasty, physiotherapy rehabilitation is a part of non-invasive treatments leading to a successful outcome after surgery. Postoperative physiotherapy, including exercises aiming at improving range of motion, muscle strengthening, achieving body balance, and gait training, was shown to improve range of motion and muscle strength of the knee [4, 5]. An European review on exercises after knee arthroplasty reported improvements in various functional outcome measures [5]. Recently, hydrotherapy has gained popularity for its use playing an important role in the rehabilitation programme after knee arthroplasty. Hydrotherapy is the external or internal use of water in any of its forms (water [liquid], ice [solid], steam [gas]) for health promotion or treatment of various diseases, at various temperatures, pressures, durations, and sites [6]. Hydrotherapy has been widely employed in various musculoskeletal and neurological conditions, from paediatric to geriatric populations [7]. Benefits of warm-bathing hydrotherapy include relieving pain and muscle spasm through warmth, reducing loads of joints through buoyancy, decreasing edema through pressure from immersion, and producing resistance to movement through turbulence and hydrostatic pressure [7, 8]. Studies proved that hydrotherapy could decrease pain, and improve physical functions, muscle strength, and quality of life in patients after total hip or knee arthroplasty [8].

To the author’s knowledge, no study examined the effect of hydrotherapy on patients after UKA (all related articles were on post-TKA patients). It is, therefore, worth investigating the effects of hydrotherapy on the clinical outcomes of patients following UKA. This was a feasibility study aiming at investigating the effects of a tailor-made hydrotherapy rehabilitation programme on improving the physical functions of patients after primary unilateral UKA. We hypothesized that the physical functions of post-UKA patients, compared with post-TKA patients, are better after the incorporation of hydrotherapy to the current joint replacement surgery physiotherapy rehabilitation.

## Methods

### Study design

This retrospective cohort study was conducted in accordance with the Declaration of Helsinki. Ethical approval was received after reviewing by the local ethics review board (Reference number: CRE2020.586). All test procedures were conducted according to the principles adopted in the Declaration of Helsinki and ICH-GCP. The study adhered to CONSORT guidelines. This study registered with clinicaltrials.gov and the trial registration number was NCT06459960.

### Patient recruitment

Patients aged 18 or above who underwent primary UKA using Oxford® Partial Knee (Zimmer Biomet, UK) at a tertiary hospital between the years 2018 and 2019 were recruited. Patients with the following conditions were excluded: (1) had major postoperative complications, (2) received revision or robotic-assisted surgery or bilateral knee arthroplasty, (3) incomplete follow-up, (4) received postoperative physiotherapy rehabilitation other than at the tertiary hospital, (5) had cognitive impairment, or (6) were unsuitable for exercise training. Comorbidities included hypertension and hyperlipidaemia. The patients included were mostly rated Grade II on ASA.

### Grouping

**Fig. 1:**
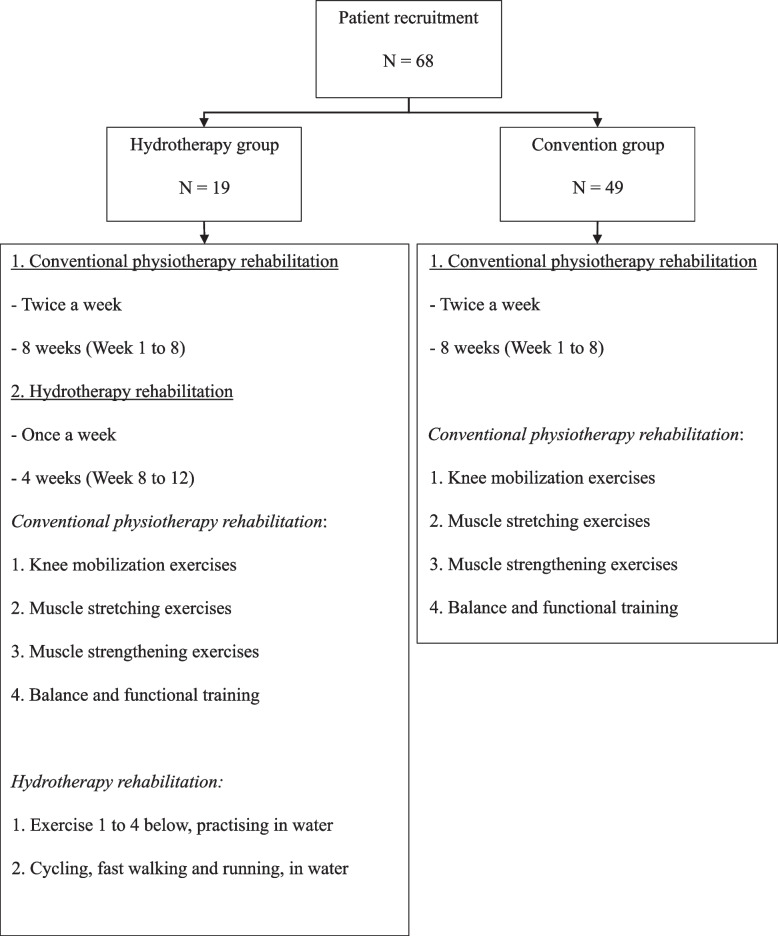
Patient recruitment, group allocation, and rehabilitation exercises involved

**Fig. 2:**
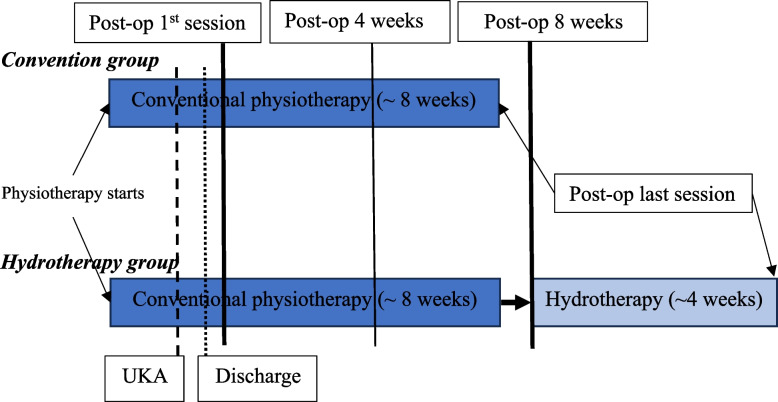
Illustration of the timeline for this feasibility study

A total of 68 patients were included and divided into (1) Hydrotherapy group (Hydrotherapy) (*n* = 19) or 2) Convention group (Convention) (*n* = 49). Figure 1 illustrates workflow of the patient recruitment. Figure 2 graphically shows the timeline of this study.

### Anaesthesia

All patients were assessed by a specialist anaesthetist to determine the optimal mode of anaesthesia. General anaesthesia or regional anaesthesia was used, depending on the clinical situation. All patients also intraoperatively received intra-articular infiltration anesthesia, which consisted of NSAIDs, adrenaline, and local anaesthetic agents.

### Walking aids used and discharge standard

Walking aid usage was based on the patients’ recovery situation, balance, and stability. All patients started using walking frames immediately following surgery. They advanced to use of walking sticks or crutches when their recovery improved. All patients achieved at least Modified Function Ambulatory Category (MFAC) IV/V before being discharged home.

### Interventions

Patients who were scheduled to have UKA received prehabilitation before surgery (about 2 months before surgery consisting of 4 sessions) as a regular regime in our total joint center (“Physiotherapy starts”, Fig. 2). Postoperative rehabilitation started at postoperative day 0 for in-patients. Most of the patients who underwent UKA were clinically fit for discharge within 3 days after surgery (“Discharge”, Fig. 2). Postoperative rehabilitation for the out-patients usually started 1–2 days after discharge. All patients finished the 8-week rehabilitation programme. Patients in the Hydrotherapy group usually started hydrotherapy treatment at postoperative 8 weeks (i.e., 2 months after UKA) and completed after 4 weeks.

#### Convention group

**Fig. 3:**
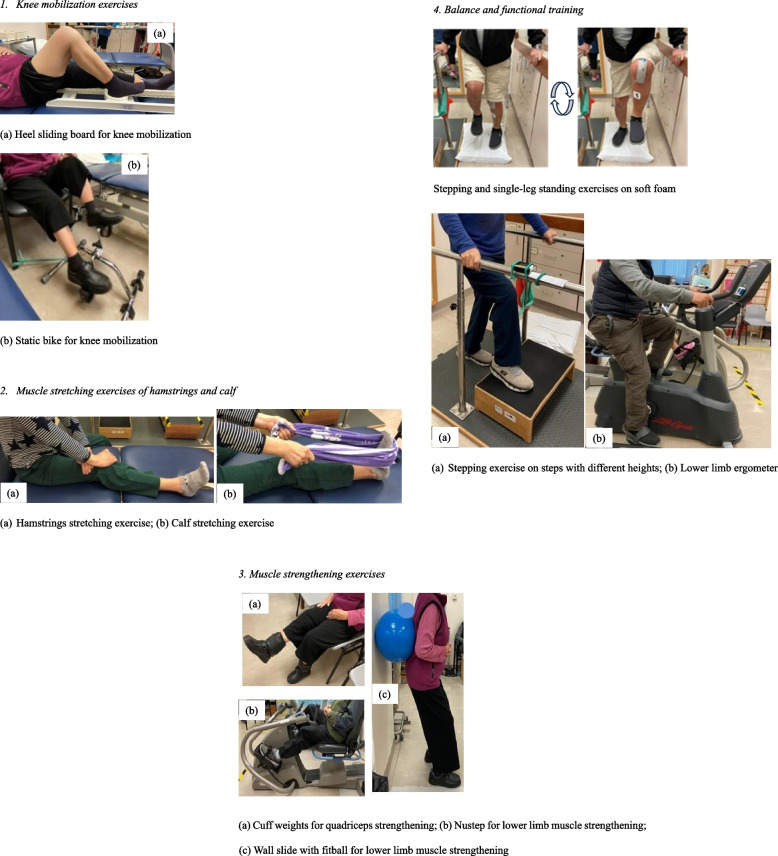
Illustrations of conventional physiotherapy rehabilitation program

The conventional physiotherapy rehabilitation program included: (1) knee mobilization exercises, such as static bike and heel sliding board, (2) muscle stretching exercises of hamstrings and calf, (3) muscle strengthening exercises, such as adding cuff weights for quadriceps strengthening and wall slide with gym ball, and (4) balance and functional training, such as stepping or single-leg standing on soft foam, stepping exercises on various heights of steps (Fig. 3 series).

To recall, physiotherapy rehabilitation program started during the perioperative period and continued after surgery twice a week, for a total of 8 weeks.

#### Hydrotherapy group

**Fig. 4:**
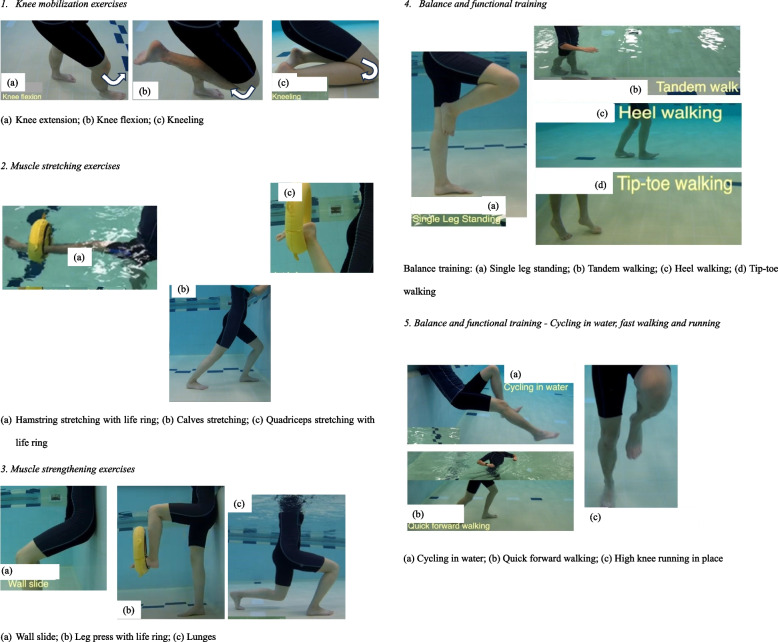
Illustrations of hydrotherapy rehabilitation program [9]

Patients practised the rehabilitation exercises in a heated pool (32 °C). Exercises included: (1) knee mobilization exercises, (2) muscle stretching exercises, (3) muscle strengthening exercises, such as wall slide, leg press with life ring, and lunges, (4) Balance and functional training: single-leg standing, tandem walking, heel walking and tip-toe walking, and (5) balance functional training: cycling in water, fast walking and running (for patients in Hydrotherapy group only) (Fig. 4).

Hydrotherapy usually started at postoperative 8 weeks (Fig. 2). Patients practised once a week for a total of four weeks, following the completion of conventional physiotherapy rehabilitation.

### Data collection time-points

Demographic characteristics were collected at patient recruitment. Outcome data were harvested by following either the clinical follow-up schedule (preoperative, postoperative 6 months and postoperative 1 year) or rehabilitation program (Post-op 1st session, Post-op 4 weeks and Post-op last session).

### Outcome assessments

#### Primary outcome

Primary outcome was Knee Society Function Score (KFS). KFS was designed to provide a simple scoring system to objectively quantify the outcomes of patients before and after TKA [10]. KFS was recorded at (1) Preoperative, (2) Postoperative 6 months, and (3) Postoperative 1 year.

#### Secondary outcomes

Two sets of secondary outcome data were collected. The 2 sets were different in terms of data-collection time points. The first set comprised: (1) Numeric pain rating scale [11] and (2) Passive range of motion—Flexion and Extension, measured using a goniometer and clinically assessed by a joint specialist [12]. Those data were taken at (1) postoperative first session, (2) Postoperative 4 weeks, and (3) postoperative last session of rehabilitation program. The second set involved: (1) self-reported walking tolerance (minutes) [13], (2) timed Up and Go Test (TUGT) (seconds) [14], and (3) a 30-s Chair Stand Test (30CST)(repetitions) [15]. Those data were collected at (1) Postoperative first session, and (2) Postoperative last session of rehabilitation program.

### Sample size calculation

The sample size in each group was determined by (1) our patient referral criteria to receive hydrotherapy and (2) Cosmin criteria [16, 17]. Medical records of our joint center showed that 1 out of 4 patients who underwent UKA met the referral criteria.

Total sample size required was calculated using G*Power 3.1.9.7. The study design was chosen as “Test of difference with two independent means (two groups)”, complementary to the following information (1) two tailed null hypothesis, (2) α = 0.05, (3) β = 0.95, and (4) power at 0.95. Effect size (= 0.94) was calculated based on the outcomes from a relevant publication on the changes in Knee Society Function Score [18]. Adding 10% of the potential drop-out rate, the calculated sample size was 68.

### Statistical analysis

All continuous data were expressed as mean and standard deviation (SD) and categorical variables as n (%), where appropriate. Basic demographics (age, sex, surgical side, body height, body weight, body mass index (BMI) between Hydrotherapy group and Convention group were compared using the Student’s *t*-test (numeric variables) or Chi-square test (categorical variables). Primary outcome (KFS) and secondary outcomes (self-reported waking tolerance, TUGT, 30CST, NPRS, and passive ROMs) were compared between Hydrotherapy group and Convention group using Student’s *t-*test. Longitudinal time-dependent comparisons of all primary and secondary outcomes mentioned were made using ANOVA. Post hoc Tukey’s honestly significant difference test was performed to correct and reduce the risk of identifying false significant differences between groups due to chance. IBM Corp. Released 2022 IBM SPSS Statistics for Windows (Version 29.0. Armonk, NY: IBM Corp) was used for data analysis. A *P* < 0.05 was considered statistically significant.

## Results

Sixty-eight patients were recruited, of which 19 patients were in hydrotherapy group (Hydrotherapy) and 49 patients were in convention group (Convention).

### Basic characteristics

Age, sex, surgical side, body height, body weight, and BMI showed no significant difference between Hydrotherapy group and Convention group (Table 1). Table 2 compares outcomes between Hydrotherapy group and Convention group.

**Table 1:** Basic demographics of the 68 patients

	**Hydrotherapy group (*n* = 19)**	**Convention group (*n* = 49)**	***P*-value**
Age (years)	67.95 ± 4.58	70.53 ± 5.85	0.089
Sex			
Male	7 (25.9) (36.8)	20 (74.1) (40.8)	1.000
Female	12 (29.3) (63.2)	29 (70.7) (59.2)	
Surgical side			
Left	9 (26.5) (47.4)	25 (73.5) (51.0)	1.000
Right	10 (29.4) (52.6)	24 (70.6) (49.0)	
Body height (cm)	158.16 ± 8.32	157.88 ± 8.14	0.900
Body weight (kg)	71.68 ± 11.14	70.06 ± 9.87	0.559
BMI (kg/m^2^)			
Mean	28.68 ± 4.15	28.06 ± 3.02	0.501
Normal^a^	0	1 (100.0) (2.0)	0.771
Overweight^a^	3 (33.3) (15.8)	6 (66.7) (12.2)	
Obese^a^	16 (27.6) (84.2)	42 (72.4) (85.7)	

^a^ BMI (Asian Standards) was used according to the WHO/IASO/IOTF. The Asia–Pacific perspective: redefining obesity and its treatment. Health communication Australia Pty Ltd.; 2000. Where BMI below 18.5 is underweight; from 18.5–22.9 is normal; from 23–24.9 is overweight; from 25–34.9 is obese

**Table 2:** Comparisons of outcomes at 3 time-points between Hydrotherapy group and Convention group

Outcomes	Hydrotherapy group (*n* = 19)	Convention group (*n* = 49)	*P*-value
Knee Society Function Score			
Preop	60.00 ± 13.33	55.61 ± 10.98	0.169
Postop 6 months	78.95 ± 14.59	70.82 ± 14.12	0.038*
Postop 1 year	83.42 ± 12.92	74.59 ± 15.34	0.030*
Numeric Pain Rating Scale			
Postop 1st session	3.95 ± 1.31	4.10 ± 1.71	0.724
Postop 4 weeks	0.95 ± 1.27	1.20 ± 1.32	0.470
Postop last session	0.00 ± 0.00	0.08 ± 0.45	0.433
Passive range of motion of knee (degrees)			
Flexion			
Postop 1st session	99.21 ± 6.51	95.10 ± 12.31	0.080
Postop 4 weeks	112.21 ± 6.55	108.16 ± 8.76	0.043*
Postop last session	118.16 ± 5.33	113.06 ± 7.76	0.011*
Extension			
Postop 1st session	2.11 ± 3.04	4.29 ± 5.40	0.102
Postop 4 weeks	0.26 ± 1.15	2.14 ± 3.06	< 0.001*
Post-op last session	0.26 ± 1.15	1.43 ± 2.70	0.015*
Self-reported walking tolerance (minutes)			
Post-op 1st session	5.26 ± 1.15	5.61 ± 2.63	0.580
Post-op 4 weeks	-	-	-
Post-op last session	71.84 ± 29.40	54.90 ± 21.37	0.011*
Timed Up and Go Test (seconds)			
Post-op 1st session	40.58 ± 10.43	50.49 ± 20.98	0.054
Post-op 4 weeks	-	-	-
Post-op last session	10.11 ± 2.33	11.43 ± 4.19	0.199
30-s Chair Stand Test (repetitions)			
Post-op 1st session	1.74 ± 2.51	1.90 ± 2.82	0.828
Post-op 4 weeks	-	-	-
Post-op last session	12.21 ± 3.41	11.47 ± 3.84	0.464

^*^Statistical significance (*P* < 0.05)

There was no significant difference in all outcomes at the first data collection time point (before operation or at postoperative 1st session) between Hydrotherapy group and Convention group. Hydrotherapy group showed significantly higher KFS than Convention group at Postoperative 6 months (*P* = 0.038) and postoperative 1 year (*P* = 0.030). Similar results were observed in the flexion and extension ROM. At Postoperative last session, patients in the Hydrotherapy group showed significantly higher walking tolerance (*P* = 0.011) compared to those in the Conventional group. No statistical significance was found in terms of NPRS, TUGT, and 30CST.

### Longitudinal comparisons

**Fig. 5:**
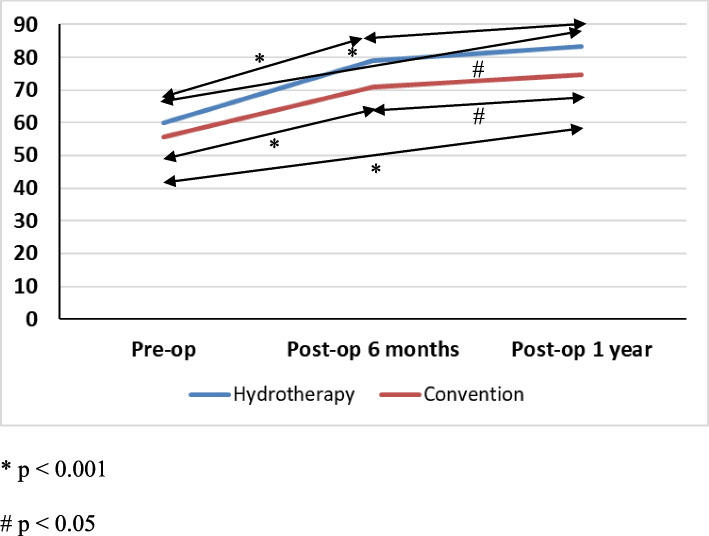
Longitudinal changes of Knee Society Function score (KFS) in Hydrotherapy group and Convention group, * *P* < 0.001, # *P* < 0.05

All outcomes showed significant improvements over time (statistically significant in all overall comparisons) (Table 3). KFS at all 3 time points were persistently higher in Hydrotherapy group than in Convention group (Fig. 5). NPRS and both flexion and extension passive ROM were significantly improved at postoperative 4 weeks and postoperative last session in both groups. Post hoc comparisons showed no statistical difference in passive ROM between postoperative 4 weeks and postoperative last session in both groups. After hydrotherapy, patients showed better outcomes than those in the convention group at the last session of self-reported walking tolerance, TUGT and 30CST.

**Table 3:** Longitudinal comparisons of outcomes in Hydrotherapy group and Convention group

**Outcomes**	**Pre-op**	**Post-op 6 months**	**Post-op 1 year**	***P*-value**
Knee Society Function Score				
Hydrotherapy group	60.00 ± 13.33	78.95 ± 14.59	83.42 ± 12.92	< 0.001*
Convention group	55.61 ± 10.98	70.82 ± 14.12	74.59 ± 15.34	< 0.001*
**Outcomes**	**Post-op 1st session**	**Post-op 4 weeks**	**Post-op last session**	***P*-value**
Numeric Pain Rating Scale				
Hydrotherapy group	3.95 ± 1.31	0.95 ± 1.27	0.00 ± 0.00	< 0.001*
Convention group	4.10 ± 1.71	1.20 ± 1.32	0.08 ± 0.45	< 0.001*
Passive range of motion of knee flexion (degrees)				
Flexion				
Hydrotherapy group	99.21 ± 6.51	112.21 ± 6.55	118.16 ± 5.33	< 0.001*
Convention group	95.10 ± 12.31	108.16 ± 8.76	113.06 ± 7.76	< 0.001*
Extension				
Hydrotherapy group	2.11 ± 3.04	0.26 ± 1.15	0.26 ± 1.15	0.007*
Convention group	4.29 ± 5.40	2.14 ± 3.06	1.43 ± 2.70	0.001*
Self-reported walking tolerance (minutes)				
Hydrotherapy group	5.26 ± 1.15	-	71.84 ± 29.40	< 0.001*
Convention group	5.61 ± 2.63	-	54.90 ± 21.37	< 0.001*
Timed Up and Go Test (seconds)				
Hydrotherapy group	40.58 ± 10.43	-	10.11 ± 2.33	< 0.001*
Convention group	50.49 ± 20.98	-	11.43 ± 4.19	< 0.001*
30-s Chair Stand Test (repetitions)				
Hydrotherapy group	1.74 ± 2.51	-	12.21 ± 3.41	< 0.001*
Convention group	1.90 ± 2.82	-	11.47 ± 3.84	< 0.001*

^*^Statistical significance (*P* < 0.05)

## Discussion

This was a feasibility study on the effect of tailor-made hydrotherapy rehabilitation on patients after UKA. The outcomes of patients who underwent UKA following hydrotherapy combined with conventional rehabilitation were compared to those who received conventional rehabilitation only. Given the baseline characteristics of patients from both groups were similar, patients in hydrotherapy group performed significantly better function 6 months and 1 year after UKA. Moreover, the range of motion, of both flexion and extension, also significantly improved at the 4th week and the last session of the rehabilitation program after UKA. Self-reported walking tolerance was found to be significantly better in Hydrotherapy group than in Convention group at the postoperative last session of rehabilitation program. NPRS, TUGT and 30CST did not show any statistical difference between these 2 groups before and after the rehabilitation program. All outcomes demonstrated significant improvements over time.

Performing an Ovid MEDLINE search on either “total knee arthroplasty” or “unicompartmental knee arthroplasty” generated the same MeSH (Medical Subject Headings) term “Arthroplasty, Replacement, Knee”, together with keyword search words (total knee arthroplasty.mp. and unicompartmental knee arthroplasty.mp.). In conjunction with the Ovid search string “hydrotherapy.mp. (“hydrotherapy” as a keyword) or Hydrotherapy/(MeSH term) retrieved the same number of articles. None of the articles discussed patients who underwent UKA. In a comparative study of a total of 100 patients who underwent TKA between the year 2008 and 2020 receiving postoperative aquatic exercise (*N* = 33), land exercise (*N* = 21), and home exercise (*N* = 46), all study groups demonstrated improved pain level and passive ROM [19]. However, statistical significance had not been reached for pain level (instrument used: VAS). Improved hip and knee muscle strength, as well as thermodynamics, hydrostatic pressure, and buoyancy (the main properties of water), might explain the pain improvement [19]. Furthermore, hydrotherapy was suggested to be introduced shortly after TKA because of the obvious joint loading reduction subsequent to pain reduction, and the benefits showed no inferiority to conventional physiotherapy alone [19]. A systematic review and meta-analysis [20] reviewing 11 randomized control trials published in 2018, and one of the study compared hydrotherapy with conventional (land-based) physiotherapy, and land-based physiotherapy alone [21]. Hydrotherapy-added groups demonstrated statistically significant improvement in activity [20]. Moreover, hydrotherapy was found to be safely delivered to patients as early as 4 days after total knee replacement surgery without increasing the risk of wound infection after applying waterproof wound dressing [22]. The results from the current study are comparable to the findings mentioned above: (1) significant improvement in pain levels and passive ROM, (2) changes in pain levels did not reach statistical significance, and (3) initiation of hydrotherapy days after surgery. The only difference is UKA vs. TKA. This is still worth further exploring the application of hydrotherapy on patients after UKA, although the percentages of patients receiving UKA remained stable, staying at 5–8% of all primary knee joint arthroplasties in the UK, substantially dependent on geographic location [2] [23]. In summary, patients who underwent UKA experienced similar advantages to those receiving TKA, with the added benefits of UKA being less invasive, requiring shorter hospital stays, and allowing for faster rehabilitation. Further randomized controlled trials or large-scale cohort studies are recommended to consolidate these observations. The results from this feasibility study provided a solid foundation for promoting further research on the application of hydrotherapy for post-UKA patients.

NPRS, TUGT, and 30CST did not show any statistical difference after adding hydrotherapy to conventional rehabilitation program. Other synthesized findings from previously cited systematic reviews and meta-analysis did not demonstrate improvements in pain and physical activity [20]. Similar results were also reported elsewhere [21]. TUGT is a sensitive and specific outcome measure primarily for determining fall risk in the elderly, also measuring balance performance [24]. TUGT cut-off of fall risk for community-dwelling elderly is 13.5 s [24], meaning that any performance of TUGT recorded longer than 13.5 s is classified as a relatively higher risk of fall, which needs follow-up assessment and management for fall prevention [24, 25]. TUGT from both groups in this study was lower than the cut-off. 30CST is a reliable outcome metric measuring muscle strength of lower limbs in elderly through performing a functional performance-based task [26]. Normative means of 30CST in the age group of 70 to 74 years (mean age in our study = 69.24) are 10.1 repetitions (female) and 11.6 repetitions (male) [27]. The mean 30CST values from our two groups were close to the normative means. Consequently, muscle strength improvements of lower limbs in patients after UKA patients after hydrotherapy resembles the effects after conventional exercises.

Several limitations of this study need to be considered. First, it was a retrospective study and selection bias could not be ruled out. Second, the small number of subjects may limit the data generalizability of this study. Third, self-reported walking tolerance, TUGT, and 30CST were not collected at postoperative 4 weeks because we aimed at comparing the data in the first and last sessions in the first place. Fourth, preoperative flexion and extension ROM was not extracted from the clinical electronic system (preoperative ROM was routinely measured as a clinic practice and the data were stored in the system) because extra resource was required to extract the data. These variables will be added to the data collection plan in further studies. Fifth, the inclusion criteria of patients receiving primary unilateral UKA with the model of Oxford® Partial Knee (Zimmer Biomet, UK) may also limit the application of research findings. This feasibility study is the first step to the further research on the effect of hydrotherapy on patients who underwent UKA. Follow-up study to support the existing findings is highly recommended. Results of this study provide insights for future studies on the effects of hydrotherapy on patients having bilateral UKA or the clinical outcomes of integrating hydrotherapy into rehabilitation. Furthermore, a randomized controlled trial with a larger sample size and similar number of subjects on each group could be another step forward.

## Conclusion

This feasibility study examined the effect of hydrotherapy on post-UKA patients. Patients who underwent UKA after hydrotherapy showed significant improvements in function and range of motion, both in flexion and extension. Additionally, walking tolerance significantly improved after completing the program. Pain, mobility, balance, and leg strength and endurance were comparable between patients with or without additional hydrotherapy, in combination with conventional physiotherapy. Since this was the first study of its kind, further research with advanced study design, larger sample size, and longer follow-up periods for patients who underwent UKA is recommended.

## Data Availability

The datasets used and/or analyzed during the current study are available from the corresponding author upon request.

## References

[1] Du H, Chen SL, Bao CD, Wang XD, Lu Y, Gu YY, et al. Prevalence and risk factors of knee osteoarthritis in Huang-Pu District, Shanghai. China Rheumatol Int. 2005;25(8):585–90. 10.1007/s00296-004-0492-7. 15309503 10.1007/s00296-004-0492-7

[2] Wilson HA, Middleton R, Abram SGF, Smith S, Alvand A, Jackson WF, et al. Patient relevant outcomes of unicompartmental versus total knee replacement: systematic review and meta-analysis. BMJ. 2019;364: l352. 10.1136/bmj.l352. 30792179 10.1136/bmj.l352 PMC6383371

[3] Ng JP, Fan JCH, Chau WW, Lau CM, Wan YC, Tse TTS, et al. Does component axial rotational alignment affect clinical outcomes in Oxford unicompartmental knee arthroplasty? Knee. 2020;27(6):1953–62. 10.1016/j.knee.2020.10.016. 33221693 10.1016/j.knee.2020.10.016

[4] McGrory BJ, Weber KL, Jevsevar DS, Sevarino K. Surgical Management of Osteoarthritis of the Knee: Evidence-based Guideline. J Am Acad Orthop Surg. 2016;24(8):e87-93. 10.5435/jaaos-d-16-00159. 27355286 10.5435/JAAOS-D-16-00159

[5] Pozzi F, Snyder-Mackler L, Zeni J. Physical exercise after knee arthroplasty: a systematic review of controlled trials. Eur J Phys Rehabil Med. 2013;49(6):877–92. 24172642 PMC4131551

[6] Mooventhan A, Nivethitha L. Scientific evidence-based effects of hydrotherapy on various systems of the body. N Am J Med Sci. 2014;6(5):199–209. 10.4103/1947-2714.132935. 24926444 10.4103/1947-2714.132935 PMC4049052

[7] Becker BE. Aquatic therapy: scientific foundations and clinical rehabilitation applications. Pm r. 2009;1(9):859–72. 10.1016/j.pmrj.2009.05.017. 19769921 10.1016/j.pmrj.2009.05.017

[8] Gibson AJ, Shields N. Effects of Aquatic Therapy and Land-Based Therapy versus Land-Based Therapy Alone on Range of Motion, Edema, and Function after Hip or Knee Replacement: A Systematic Review and Meta-analysis. Physiother Can. 2015;67(2):133–41. 10.3138/ptc.2014-01. 25931664 10.3138/ptc.2014-01 PMC4407119

[9] Physiotherapy_Department_Alice_Ho_Miu_Ling_Nethersole_Hospital: Hip/Knee Joint Replacement: Hydrotherapy. https://www3.ha.org.hk/AHNH/content/physio/physio_chi/e_resource/TJR_Mainpage_chi/TJR_Hydro/Staticpage/TJR_Hydro_static.htm (2021). Accessed 22 February 2024.

[10] Insall JN, Dorr LD, Scott RD, Scott WN. Rationale of the Knee Society clinical rating system. Clin Orthop Relat Res. 1989;248:13–4. 2805470

[11] Lampropoulou S, Nowicky AV. Evaluation of the numeric rating scale for perception of effort during isometric elbow flexion exercise. Eur J Appl Physiol. 2012;112(3):1167–75. 10.1007/s00421-011-2074-1. 21769733 10.1007/s00421-011-2074-1

[12] Klein LJ. 5 - Evaluation of the Hand and Upper Extremity. In: Cooper C, editor. Fundamentals of Hand Therapy. 2nd ed. St. Louis: Mosby; 2014. p. 67–86.

[13] Wallis JA, Webster KE, Levinger P, Singh PJ, Fong C, Taylor NF. The maximum tolerated dose of walking for people with severe osteoarthritis of the knee: a phase I trial. Osteoarthritis Cartilage. 2015;23(8):1285–93. 10.1016/j.joca.2015.04.001. 25882926 10.1016/j.joca.2015.04.001

[14] Bade MJ, Kohrt WM, Stevens-Lapsley JE. Outcomes before and after total knee arthroplasty compared to healthy adults. J Orthop Sports Phys Ther. 2010;40(9):559–67. 10.2519/jospt.2010.3317. 20710093 10.2519/jospt.2010.3317 PMC3164265

[15] Millor N, Lecumberri P, Gómez M, Martínez-Ramírez A, Izquierdo M. An evaluation of the 30-s chair stand test in older adults: frailty detection based on kinematic parameters from a single inertial unit. J Neuroeng Rehabil. 2013;10:86. 10.1186/1743-0003-10-86. 24059755 10.1186/1743-0003-10-86 PMC3735415

[16] Mokkink LB, Terwee CB, Knol DL, Stratford PW, Alonso J, Patrick DL, et al. Protocol of the COSMIN study: COnsensus-based Standards for the selection of health Measurement INstruments. BMC Med Res Methodol. 2006;6:2. 10.1186/1471-2288-6-2. 16433905 10.1186/1471-2288-6-2 PMC1368990

[17] Mokkink LB, Terwee CB, Patrick DL, Alonso J, Stratford PW, Knol DL, et al. The COSMIN checklist for assessing the methodological quality of studies on measurement properties of health status measurement instruments: an international Delphi study. Qual Life Res. 2010;19(4):539–49. 10.1007/s11136-010-9606-8. 20169472 10.1007/s11136-010-9606-8 PMC2852520

[18] Lee HJ, Park YB, Song MK, Kwak YH, Kim SH. Comparison of the outcomes of navigation-assisted revision of unicompartmental knee arthroplasty to total knee arthroplasty versus navigation-assisted primary TKA. Int Orthop. 2019;43(2):315–22. 10.1007/s00264-018-4028-2. 29916003 10.1007/s00264-018-4028-2

[19] Lee CH, Kim IH. Aquatic Exercise and Land Exercise Treatments after Total Knee Replacement Arthroplasty in Elderly Women: A Comparative Study. Medicina (Kaunas). 2021;57(6). 10.3390/medicina57060589. 10.3390/medicina57060589 PMC8229167 34201120

[20] Henderson KG, Wallis JA, Snowdon DA. Active physiotherapy interventions following total knee arthroplasty in the hospital and inpatient rehabilitation settings: a systematic review and meta-analysis. Physiotherapy. 2018;104(1):25–35. 10.1016/j.physio.2017.01.002. 28802773 10.1016/j.physio.2017.01.002

[21] Rahmann AE, Brauer SG, Nitz JC. A specific inpatient aquatic physiotherapy program improves strength after total hip or knee replacement surgery: a randomized controlled trial. Arch Phys Med Rehabil. 2009;90(5):745–55. 10.1016/j.apmr.2008.12.011. 19406293 10.1016/j.apmr.2008.12.011

[22] Villalta EM, Peiris CL. Early aquatic physical therapy improves function and does not increase risk of wound-related adverse events for adults after orthopedic surgery: a systematic review and meta-analysis. Arch Phys Med Rehabil. 2013;94(1):138–48. 10.1016/j.apmr.2012.07.020. 22878230 10.1016/j.apmr.2012.07.020

[23] Di Martino A, Bordini B, Barile F, Ancarani C, Digennaro V, Faldini C. Unicompartmental knee arthroplasty has higher revisions than total knee arthroplasty at long term follow-up: a registry study on 6453 prostheses. Knee Surg Sports Traumatol Arthrosc. 2021;29(10):3323–9. 10.1007/s00167-020-06184-1. 32740877 10.1007/s00167-020-06184-1 PMC8458185

[24] Shumway-Cook A, Brauer S, Woollacott M. Predicting the probability for falls in community-dwelling older adults using the Timed Up & Go Test. Phys Ther. 2000;80(9):896–903. 10960937

[25] Benavent-Caballer V, Sendín-Magdalena A, Lisón JF, Rosado-Calatayud P, Amer-Cuenca JJ, Salvador-Coloma P, et al. Physical factors underlying the Timed “Up and Go” test in older adults. Geriatr Nurs. 2016;37(2):122–7. 10.1016/j.gerinurse.2015.11.002. 26707544 10.1016/j.gerinurse.2015.11.002

[26] Jones CJ, Rikli RE, Beam WC. A 30-s chair-stand test as a measure of lower body strength in community-residing older adults. Res Q Exerc Sport. 1999;70(2):113–9. 10.1080/02701367.1999.10608028. 10380242 10.1080/02701367.1999.10608028

[27] Macfarlane DJ, Chou KL, Cheng YH, Chi I. Validity and normative data for thirty-second chair stand test in elderly community-dwelling Hong Kong Chinese. Am J Hum Biol. 2006;18(3):418–21. 10.1002/ajhb.20503. 16634026 10.1002/ajhb.20503

